# Clustering of Diamond Nanoparticles, Fluorination and Efficiency of Slow Neutron Reflectors

**DOI:** 10.3390/nano11081945

**Published:** 2021-07-28

**Authors:** Aleksander Aleksenskii, Markus Bleuel, Alexei Bosak, Alexandra Chumakova, Artur Dideikin, Marc Dubois, Ekaterina Korobkina, Egor Lychagin, Alexei Muzychka, Grigory Nekhaev, Valery Nesvizhevsky, Alexander Nezvanov, Ralf Schweins, Alexander Shvidchenko, Alexander Strelkov, Kylyshbek Turlybekuly, Alexander Vul’, Kirill Zhernenkov

**Affiliations:** 1 Ioffe Institute, Polytechnicheskaya Str. 26, 194021 St. Petersburg, Russia; blin@mail.ioffe.ru (A.A.); Dideikin@mail.ioffe.ru (A.D.); Avshvid@mail.ioffe.ru (A.S.); AlexanderVul@mail.ioffe.ru (A.V.); 2 National Institute of Standards and Technology, Gaithersburg, MD 20899, USA; markus.bleuel@nist.gov; 3 Department of Materials Science and Engineering, University of Maryland, College Park, MD 20742, USA; 4 European Synchrotron Radiation Facility, 71 av. des Martyrs, F-38042 Grenoble, France; alexei.bossak@esrf.fr (A.B.); aleksandra.chumakova@esrf.fr (A.C.); 5 Institut de Chimie de Clermont-Ferrand (ICCF UME 6296), Université Clermont Auvergne, CNRS, 24 av. Blaise Pascal, F-63178 Aubière, France; marc.dubois@uca.fr; 6 Department of Nuclear Engineering, North Carolina State University, Raleigh, NC 27695, USA; ekorobk@ncsu.edu; 7 Joint Institute for Nuclear Research, 6 Joliot Curie, 141980 Dubna, Russia; lychag@nf.jinr.ru (E.L.); muz@nf.jinr.ru (A.M.); grigorijnekhaev@yandex.ru (G.N.); nezvanov@jinr.ru (A.N.); str@jinr.ru (A.S.); kilishbek-t@mail.ru (K.T.); k.zhernenkov@fz-juelich.de (K.Z.); 8 Faculty of Physics, Lomonosov Moscow State University, GSP-1, Leninskie Gory, 119991 Moscow, Russia; 9 Department of Nuclear Physics, Dubna State University, Universitetskaya 19, 141982 Dubna, Russia; 10 Institute Max von Laue–Paul Langevin, 71 av. des Martyrs, F-38042 Grenoble, France; schweins@ill.eu; 11 Faculty of Physics and Technology, L.N. Gumilyov Eurasian National University, Satpayev Str. 2, Nur-Sultan 010000, Kazakhstan; 12 The Institute of Nuclear Physics, Ministry of Energy of the Republic of Kazakhstan, Ibragimova Str. 1, Almaty 050032, Kazakhstan; 13 JCNS at Heinz Maier-Leibnitz Zentrum (MLZ), Forshungzentrum Julich GmbH, 1 Lichtenbergstrasse, G-85748 Garching, Germany

**Keywords:** detonation nanodiamonds, nanopowder, reflectors of slow neutrons, albedo, clustering and agglomeration of nanodiamonds, deagglomeration, fluorination, size distribution, Monte Carlo

## Abstract

Neutrons can be an instrument or an object in many fields of research. Major efforts all over the world are devoted to improving the intensity of neutron sources and the efficiency of neutron delivery for experimental installations. In this context, neutron reflectors play a key role because they allow significant improvement of both economy and efficiency. For slow neutrons, Detonation NanoDiamond (DND) powders provide exceptionally good reflecting performance due to the combination of enhanced coherent scattering and low neutron absorption. The enhancement is at maximum when the nanoparticle diameter is close to the neutron wavelength. Therefore, the mean nanoparticle diameter and the diameter distribution are important. In addition, DNDs show clustering, which increases their effective diameters. Here, we report on how breaking agglomerates affects clustering of DNDs and the overall reflector performance. We characterize DNDs using small-angle neutron scattering, X-ray diffraction, scanning and transmission electron microscopy, neutron activation analysis, dynamical light scattering, infra-red light spectroscopy, and others. Based on the results of these tests, we discuss the calculated size distribution of DNDs, the absolute cross-section of neutron scattering, the neutron albedo, and the neutron intensity gain for neutron traps with DND walls.

## 1. Introduction

Neutrons are used in numerous scientific research disciplines to probe samples and their interactions over a broad range of energy and length scales, but they are also studied by themselves to examine fundamentals of this universe by probing the laws of nature. Major efforts all over the world are devoted to improving the intensity of sources of neutrons and the efficiency of delivery of neutrons to experimental installations. This is equally relevant for nuclear research reactors, neutron spallation sources, compact accelerator-driven neutron sources, and others. In this context, neutron reflectors play a key role because they improve the performance of neutron sources and delivery systems in an economical and efficient way.

The principle of slow neutron reflection from NanoDiamond (ND) powders is the enhanced coherent scattering of neutrons from the NDs. Very cold neutrons (VCNs) are reflected from ND powders diffusively at all incidence angles (such diffusive reflectivity is called neutron albedo) [1,2,3,4], and cold neutrons (CNs) are reflected quasi-specularly, provided the incidence angles are small.

ND powders can be produced either with lasers [5] or using detonation (Detonation NanoDiamonds, DNDs). In this study, we focus on DNDs because only they are currently available in the large amounts needed for the production of full-scale neutron reflectors. DNDs [6,7], with sizes of 4–5 nm, provide an exceptionally good performance, as they combine such properties as the large length of coherent scattering of carbon (C) and the low losses of neutrons. The length of coherent scattering is 6.65 fm, and the cross-section of coherent scattering is 5.55 b. Possible channels of neutron losses are neutron absorption and inelastic scattering (the gained energy typically results in the neutron acceleration beyond the VCN range). The cross-section of absorption is 3.5 mb, and the cross section of inelastic scattering is low and changes as a function of temperature.

While diamond bulk by itself introduces small neutron losses, the enormous surface area of the DNDs introduces the possibility of contaminations, which are much less neutron-friendly. Mostly hydrogen (H) in raw DNDs causes losses of neutrons and reduces the reflection efficiency. Reduction of the H impurities by the fluorination of DNDs improves the probability of neutron quasi-specular reflection [8]. Additionally, further measurements of VCN albedo from such designed Fluorinated DND (F-DND) powders are planned.

In raw DNDs, the H fraction is large (one H per 7.4 ± 0.2 C atoms). Here and below, the uncertainty bars correspond to one standard deviation. The cross section of absorption of neutrons by H is large (0.33 b), and the cross-section of incoherent scattering is very large (108 ± 2 b at room temperature [9]). The later consists of inelastic and elastic parts; their ratio changes as a function of temperature. H atoms in DNDs are bound as C-OH, C-H, CH_2_, and COOH [10,11]. In previous studies, we reduced, by the chemical treatment of DNDs in F_2_ gas [12,13,14], the fraction of H by at least ~30 times. In freshly prepared F-DNDs, one H atom is present per 430 ± 30 C atoms. Alternative approaches would be to reduce the fraction of H by deuteration or modification of conditions of its production [15]. However, we focus on fluorination due to its efficiency and universality.

DNDs are particularly interesting for improving performance of VCN and CN sources as well as for the loss-limited extraction of slow neutrons from high-radiation zones. This argument motivates further measurements of DND neutron-scattering cross-sections [16,17,18] and the inclusion of neutron scattering on DNDs to neutron simulations [18,19,20]. These cross-section studies showed the smallness of inelastic scattering and the importance of accounting for clustering and agglomeration of DNDs in the interpretation of neutron-scattering experiments. However, we have to distinguish clusters and agglomerates. Agglomerates assume stronger bonding between DNDs, while clustering is the most general term and represents only the close packing of neighboring DNDs without any relation to the degree of their bonding. It is clusters that are of importance for neutron scattering and neutron reflectors, while different methods of characterization of DND samples show different sensitivity to the strength of DND bonding.

The enhanced coherent scattering of slow neutrons on the nanoparticle is most efficient when the DND diameter is close to the neutron wavelength. Thus, the mean size, shape, and the size distribution of DNDs are important [21]. Agglomeration increases the effective diameter of DNDs, and therefore, it is relevant. Here, we investigate the effect of breaking agglomerates of DNDs on the clustering and overall reflector performance. We use small quantities of F-DND and Deagglomerated F-DND (DF-DND) samples, ~50 mg. Significantly larger amounts of optimized DNDs will be produced later for building real-scale neutron reflectors.

The overall reflector performance also depends on particular applications and geometries [22,23]. Therefore, we have simulated some particular cases where the effect of breaking clusters in DND powders is most important.

In Section 2, we describe DND samples and justify our choice of experimental methods for their characterization as well as the model used for the simulation of neutron diffusion in DND powders. In Section 3, we present the experimental methods, including scanning electron microscopy (SEM), transmission electron microscopy (TEM), dynamic light scattering (DLS), neutron-activation analysis (NAA), infra-red (IR) spectroscopy, X-ray diffraction (XRD), and small-angle neutron scattering (SANS). Based on these data, in Section 4, we discuss the calculated size distribution of DNDs, the absolute cross-section of neutron scattering, the neutron albedo, and the neutron intensity gain for neutron traps with DND walls.

## 2. Materials and Methods

### 2.1. Samples

DND powders are obtained by the detonation synthesis. They contain particles with a diamond crystal core of 4–5 nm. Numerous researches underlined the narrow size distribution of DNDs [10,24,25,26]. Ref. [27] assumed that a thermodynamically stable form of nanocarbon at such sizes is diamond rather than graphite. This hypothesis has been confirmed by the simulations of stable clusters of carbon [28].

A core-shell model [7] is usually used to describe the structure of DND particles. The diamond core is assumed to have sp^3^ hybridization, and it has the polyhedron shape [29]. A non-crystalline C shell has sp^3^-sp^2^ hybridization of C atoms and surrounds the core. C-H_x_, C-C-H, C-OH, C-O, C=C are present in the shell [11]. The shell thickness is 0.4–1.0 nm [30,31,32]. Industrial DND powder contains strongly connected, most dense agglomerates (agglutinates) with sizes of 40–200 nm consisting of 4–5 nm individual particles. DND powders can be deagglomerated. One process is based on mechanical milling [33,34]. Another one uses annealing in different gases [35,36]. DNDs in commercially available powders have a hierarchical structure of agglomerates [34,37,38]. The sizes of larger agglomerates are above 1 μm; they are more easily destroyed [39].

For compatibility with the previous results [8,12,13,14,40], we have used equivalent DNDs from the Federal State Unitary Enterprise, “Russian Federal Nuclear Center—Academician E.I. Zababakhin All-Russian Research Institute of Technical Physics” (FSUE “RFNC-VNIIF”), Snezhinsk, Russia; Technical Regulations TY 2-037-677-94.

To reduce chemically bonded H contaminations, raw DNDs were fluorinated at the Université Clermont Auvergne, Aubière, France, as described in ref. [8]. During fluorination, whereas sp^3^ diamond carbons (the DND cores) are non-reactive towards F_2_ gas, two reactions compete for the sp^2^ carbon shell at the reaction temperature of 450 °C in pure F_2_ gas (pressure of 1 atm): fluorination and decomposition. During fluorination, the hybridization of C changes from sp^2^ to sp^3^, and covalent C-F bonds are formed. We denote them C_ex_-_sp_^2^-F. During decomposition, CF_4_ and C_2_F_6_ gases are formed. The treatment conditions are chosen so as to favor the decomposition. Diamond cores are not affected by F_2_ molecules because of the stabilization of C atoms in the sp^2^ hybridization and in the crystalline C lattice. Besides, F from covalent C-F bonds can substitute H atoms bonded to sp^3^ C in CH, CH_2_, or C-OH groups.

Another source of H in DNDs is the absorption of water from air moisture, as raw powders are very hygroscopic. Thus, the quantity of H increases twofold [9] in the air with ~60% humidity. Raw DNDs are hydrophilic because of –OH and –COOH groups on the surface. It was shown that hard fluorination [8], which replaces H by F, also increases hydrophobicity, further reducing possible H contamination.

A fraction of the F-DND powder was further deagglomerated at the Ioffe Institute, St. Petersburg, Russia, using a method similar to that proposed in [36,41]. Dispersing of F-DND in ethanol is known to form a stable colloidal solution [42]. For this purpose, 2 g of F-DND powder was dispersed in 200 mL of 96% ethanol by sonication (the frequency of 22 kHz, the power of 0.7 W/g, and the time of 15 min). The resulting F-DND solution was centrifuged (in a Sigma 6-16 centrifuge with the acceleration a_max_ = 1.8 × 10^4^ g for 40 min) in order to separate particles in ethanol by size. The supernatant containing deagglomerated F-DND particles with sizes of ~5 nm was carefully separated from the sediment. The yield of deagglomerated particles was 20%; it is estimated as the ratio of their mass after ethanol removal to the initial mass of the F-DND powder. Ethanol was removed in two stages: (1) drying of supernatant in a rotary evaporator and (2) heating of wet powder in air at the temperature of 300 °C for 1 h. We call the resulting powder DF-DND, where DF means that the sample was first fluorinated and then deagglomerated.

### 2.2. Rationale for the Choice of Experimental Methods

In order to characterize these F-DND and DF-DND samples, we used several complementary techniques.

SEM and TEM images allow the direct visualization of clusters in F-DND samples and their decreased probability in DF-DND samples. They allow measuring DND sizes (cores + shells) as well as sizes of DND structures. On the other hand, precise quantitative treatment of SEM and TEM data is difficult because it would require an analysis of many images in order to get sufficient statistics. In addition, the question remains whether these images are fully representative for the whole sample and not just for individual areas. Examples of SEM and TEM images are given in Section 3.1.

The DLS method is well representative for the whole sample and is used to prove the effect of deagglomeration of F-DNDs. It probes the motion of DNDs and the agglomerates in liquid dispersion. One should note also that clustering of DNDs immersed in liquid might differ from clustering in dry powder. Additionally, clustering of DNDs in dry powder after deagglomeration might differ from the clustering in liquid after deagglomeration. We present results of DLS studies in Section 3.2.

NAA and IR spectroscopy are useful because the chemical composition of DNDs can affect neutron scattering and losses. The thicker the neutron reflector layer, the more important becomes the knowledge about chemical composition in simulations of neutron albedo. As was shown previously in [8], F-DND samples are quite clean from the neutron absorbing impurities; therefore, the goal of the present work was rather to verify that large, new impurities were not introduced by the deagglomeration process. Results of chemical analysis of the samples are presented in Section 3.3.

SANS results are unambiguous. They are directly used for the simulation of neutron scattering in DND powders. SANS measure both individual DNDs and the clusters. SANS data are given in Section 3.5. In order to extrapolate the results to other neutron velocities, we applied the model of discrete-sized diamond nanospheres, which was introduced in [40] and mentioned briefly in Section 2.3. A more detailed description of the method will be published elsewhere.

XRD is sensitive to the crystal structure of DNDs. It is useful to verify that the deagglomeration procedure does not affect the sizes of crystalline cores of DNDs and sp^2^ shells; otherwise, there would be two contributions to the change in SANS results, and it would be more difficult to interpret the data. Results of XRD measurements are given in Section 3.4.

### 2.3. The Model of Discrete-Sized Diamond Nanospheres

This model was introduced in [40] to simulate neutron scattering on DNDs and the clusters. It substitutes an actual powder sample (with a continuous set of DND radii) with a discrete set of nanosphere radii. This representation is similar to the expansion of an arbitrary mathematical function into a series. Scatterers populations in the radius bins are chosen to describe the measured data. This model provides a good and simple approximation to calculate the neutron diffusion in DND powders.

The approach we propose allows using both approximate and precise quantum mechanical methods for calculating the cross-sections of elastic coherent scattering of neutrons on nanospheres [43]. Precise methods provide an accurate size distribution of scatterers. Approximate methods provide faster calculations in the range of their applicability depending on sizes of scatterers and neutron energies, but they misrepresent the size distributions beyond this range; however, such misrepresentations do not affect simulations of neutron transport.

One should note that the density of real clusters differs from that of diamond. Additionally of importance is that the forms of both clusters and DNDs are not spherical. Therefore, cross-sections of neutron scattering on real, loose clusters are below those on solid nanospheres. As a result, this model underestimates the fraction of clusters and DNDs proportionally to the difference in scattering cross-sections. Thus, the mass of nanospheres (it is called the effective mass in our model) is smaller than the powder mass. This difference is explained by the effects of C in the sp^2^-phase, other than C elements, the presence of pores, the real shapes of DND cores, the interference effects, etc.

## 3. Results

### 3.1. SEM and TEM

We measured SEM images with a microscope Hitachi SU8020 (JINR, Dubna) with a cold field cathode. DNDs were attached with silver glue to an aluminum plate and coated with a ~20 nm layer of gold-palladium alloy; the coating provided electrical conductivity.

**Figure 1:**
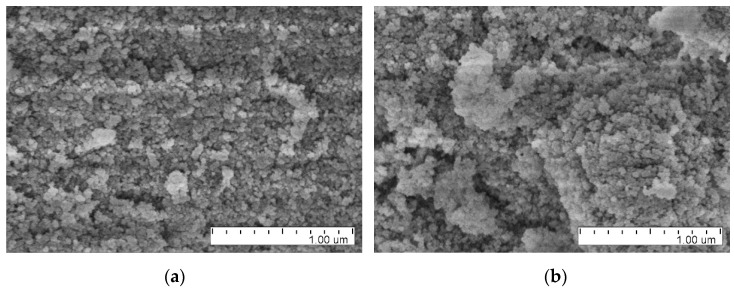

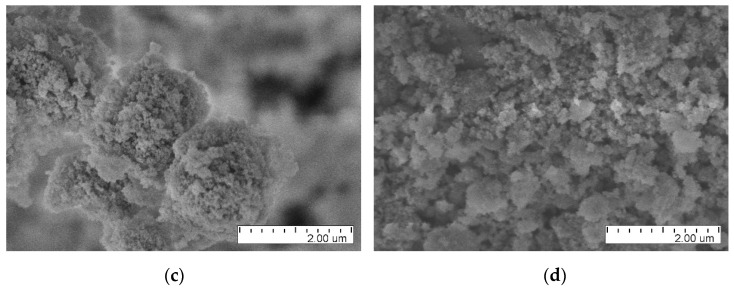
Examples of SEM images: (**a**,**b**) DF-DNDs; (**c**,**d**) F-DNDs.

Figure 1 reveals clusters in all samples; we show only four images of the many available. It is difficult to clearly conclude on the change in powder structure caused by deagglomeration. However, SEM images reveal a lower amount of agglomerates in DF-DND samples.

**Figure 2:**
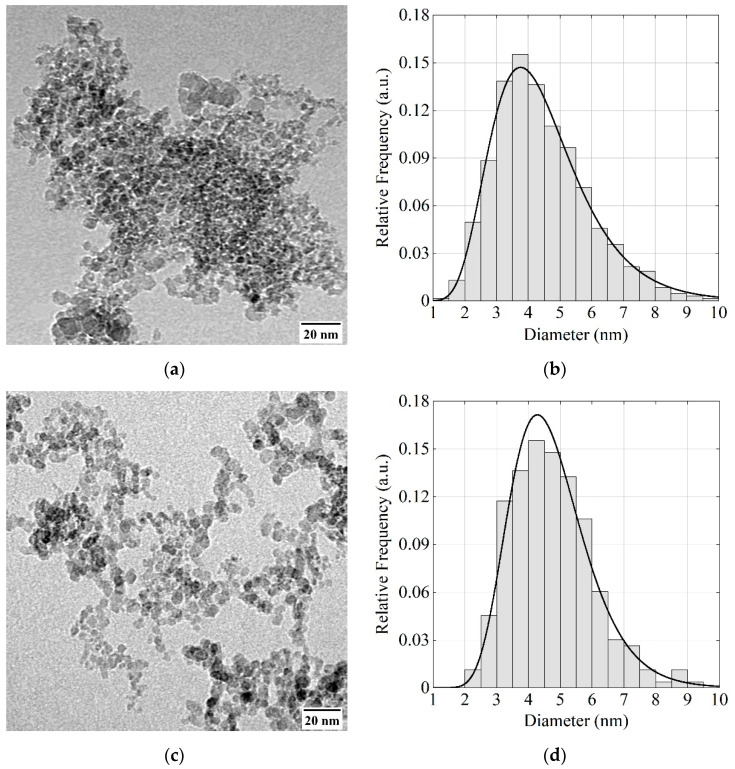
(**a**,**c**) TEM images of the F-DND and DF-DND samples; (**b**,**d**) respective diameter distributions of the DNDs. Black solid lines correspond to the lognormal distribution.

In Figure 2, one can see an image of F-DNDs measured using a TEM (FEI Tecnai G2 30 S-TWIN, NRC “Kurchatov Institute”—CRISM “Prometey”, St. Petersburg, Russia) and a corresponding distribution of DND sizes. The procedure is as described in [40].

The mean diameter of DND primary particles was evaluated using the data shown in Figure 2. It is 4.6 ± 0.1 nm for both F-DNDs and DF-DNDs. Moreover, the size distribution shapes are the same. However, the density of agglomerates seems to decrease after deagglomeration.

### 3.2. DLS

**Figure 3:**
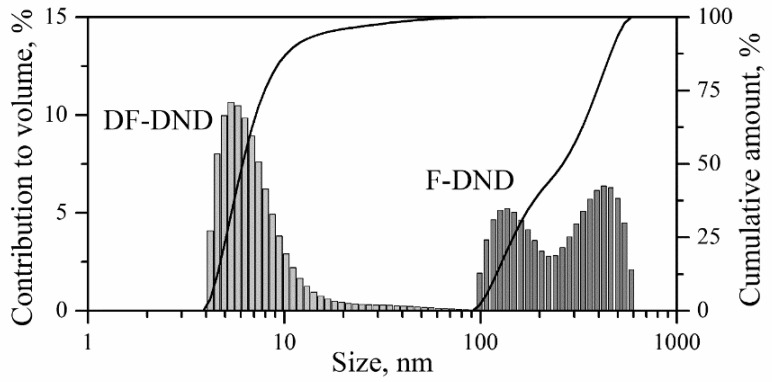
Size distributions in F-DNDs and DF-DNDs in ethanol measured by the DLS method.

Size distributions of F-DNDs and DF-DNDs dispersed in ethanol were measured using the DLS method with an analyzer Litesizer 500 (Anton Paar GmbH, Graz, Austria). F-DNDs mainly form agglomerates with sizes of 100–600 nm (F-DND in Figure 3). The agglomerates have a bimodal size distribution with maxima at 130 nm and 420 nm. Centrifugation of a colloidal solution with F-DNDs allowed to settle most of the agglomerates. Separate 5.5 nm F-DNDs were observed by DLS in the supernatant extracted after the centrifugation (DF-DND in Figure 3). It is worth emphasizing that the F-DND suspension prepared by strong sonication contains deagglomerated particles, but the presence of agglomerates does not allow them to be detected by the DLS method [44]. Individual particles could be detected only after removing a greater part of agglomerates from the suspension.

### 3.3. Chemical Composition by NAA and IR Spectroscopy

Impurities in DNDs can significantly affect the efficiency of neutron albedo. First, they can cause extra losses of neutrons during their diffusion in DNDs because of the neutron absorption and inelastic scattering. Second, a large amount of impurities can even change the shape of the DND optical potential that would lead to a change in the neutron scattering cross-sections. Third, impurities could affect the agglomeration process. Fourth, impurities can be activated in strong ionizing fields, thus complicating manipulations with such materials.

We have carried out many studies of impurities in the original DNDs, F-DNDs, and powders at various stages of the deagglomeration process of the original DNDs using neutron activation analysis, prompt-γ neutron analysis at the ILL high-flux reactor [45], and the IBR-2 reactor [46], X-ray photoelectron spectroscopy, and Raman light scattering.

In summary, our results show that only H (before fluorination), F (after fluorination), O, and N are present in DNDs in the amounts that could change the neutron-scattering properties. We found that the fraction of chemically bonded H atoms in initial DNDs is below ~8% of C atoms. Thus, they could form a layer with a thickness of 1–2 atoms on the DND surface. Since H has a scattering length of opposite sign than that of C, it could potentially deform the shape of the neutron optical potential of DND (the potential is proportional to the scattering length). During fluorination, at least ~97% of H atoms are replaced by F atoms [12]. The coherent scattering length of F differs by ~30% from that of C. However, the fact that the substitution of H by F did not affect the neutron elastic scattering is evidenced by the scattering curves for DNDs and F-DNDs [40].

Our conclusion (from studies on non-fluorinated samples) strongly supports that deagglomeration does not increase the amount of non-H impurities sufficient to affect neutron elastic scattering. Consequently, our DF-DNDs sample was characterized solely with IR spectroscopy sensitive only to H.

According to the results of Raman scattering, the fraction of H does not increase during the F-DNDs deagglomeration. Therefore, we are confident that a possible change in impurities during deagglomeration cannot be so large as to affect the neutron scattering on DNDs.

In all studied powders and after various stages of their deagglomeration and chemical treatment, the fraction of N is 0.4–2.0%, which corresponds to the data available in the literature indicating that N is located inside diamond crystals in the form of impurity atoms [47].

The total fraction of other impurities does not exceed 0.05 at. % and cannot significantly affect the neutron elastic scattering studied by SANS in the present paper. However, one should note that they could affect neutron albedo from a thick layer of DNDs due to an increase of the neutron loss rate. The issue of producing DNDs without impurities of N, B, Cl, and metals or deeply purifying DNDs from these impurities remains an important task but is beyond the scope of this study.

The composition of surface functional groups was analyzed using IR spectroscopy. IR spectra of diffusive scattering from F-DNDs and DF-DNDs were recorded in 100 measurements with the resolution of 4 cm^−1^ using an Infralum FT-08 spectrometer with Fourier transform (CG “Lumex”, Russia).

**Figure 4:**
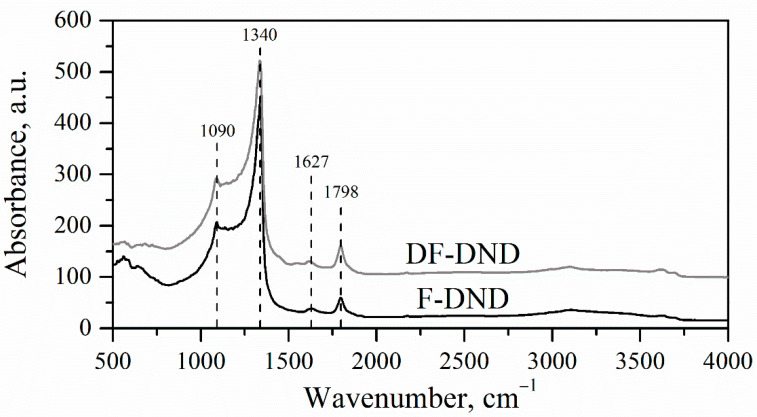
IR spectra of F-DNDs and DF-DNDs.

IR spectra of DND powders (see Figure 4) show characteristic absorption bands associated with vibrations of CF_x_ groups in addition to the narrow band of C-C vibration for diamond core (1340 cm^−1^). Bands between 1090 and 1220 cm^−1^ correspond to CF_2_ and CF_3_ symmetric stretch, CF_2_ asymmetric stretch, as well as CF=CF_2_, CF_3_-CF_2_ and CF stretch vibrations [48]. This latter contribution dominates according to solid-state NMR characterization [12]; CF_2_ and CF_3_ are not evidenced neither by ^19^F nor ^13^C measurements. C-F bonds result from both conversion of C-H and COH into C-F in the diamond core and fluorination of sp^2^ C in the shell (C_ex_-_sp_^2^-F). O-H bending and stretching in molecules of adsorbed water corresponds to the weak absorption peak at 1627 cm^−1^ and a wide band between 2750 and 3750 cm^−1^. The peak at 1798 cm^−1^ is probably due to C=O stretching. Dangling bonds are formed during fluorination by cleavage of C-C, C-O, and C-H bonds. Some of them react with O_2_ and moisture in air after exposure to air after fluorination. We can conclude that IR spectra stay virtually unchanged by the deagglomeration procedure.

### 3.4. XRD

**Figure 5:**
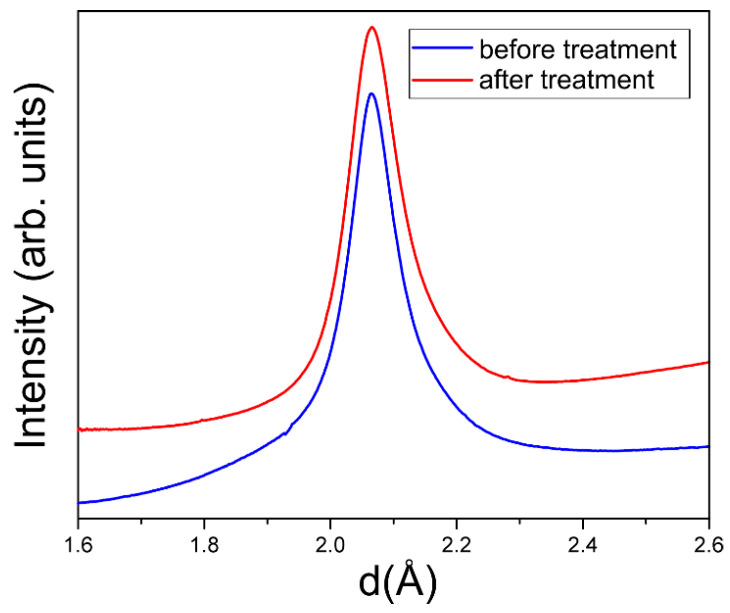
Profile of 111 diamond diffraction peak before and after the deagglomeration treatment. Graphs are shifted along the vertical axis for the visibility.

X-ray diffraction was measured at the ID28 instrument at ESRF with PILATUS3 1M area detector with X-ray wavelengths of 0.697 Å and 0.980 Å. DNDs were inserted in quartz capillaries with the diameter of 200 μm. Data were evaluated using SNBL Toolbox [49,50] software. No significant difference between the peak shape before and after deagglomeration treatment is observed, as shown in Figure 5, in line with the expectations.

### 3.5. SANS

We characterized F-DND and DF-DND samples using four different SANS instruments. Two of them are D11 at ILL [51,52,53] and YuMO time-of-flight spectrometer in the two-detector mode at FLNP, JINR [54]. At D11, the neutron wavelength and the range of transferred momenta (Q) were 6 Å and 10^−2^ nm^−1^ < Q < 10^0^ nm^−1^. At YuMO, these were 0.7–5.0 Å and 7·10^−2^ nm^−1^ < Q < 10^1^ nm^−1^. Both samples were studied at each of the instruments mentioned above. Two other instruments are NGB30 and NG7 at the NIST Center for Neutron Research [55]. F-DNDs were measured only at NGB30, and DF-DNDs were measured only at NG7. At NGB30, the neutron wavelength and the range of transferred momenta were 6 Å and 3.4·10^−2^ nm^−1^ < Q < 1.2·10^0^ nm^−1^. At NG7, these were 6 Å and 3.5·10^−2^ nm^−1^ < Q < 6.0·10^0^ nm^−1^. We calibrated the absolute SANS intensity for both samples using the transmission data obtained at NGB30 and NG7. This absolute calibration has a critical importance for the simulation of neutron transport in the powders. As usual, the correction for background scattering from empty cuvettes was applied. The SANS data obtained at NGB30 and NG7 were evaluated using the Igor macros [56].

We put samples inside Quartz SUPRASIL 1-mm cells at D11, NGB30, and NG7 and in dur-aluminum 1-mm cells, with 1-mm windows, at YuMO. The density of F-DND samples varied from 0.24 ± 0.01 to 0.37 ± 0.01 g/cm^3^ at D11. It was 0.36 ± 0.01 g/cm^3^ at YuMO and 0.19 ± 0.01 g/cm^3^ at NGB30. The density of the DF-DND sample was equal to 0.56 ± 0.01 g/cm^3^ at NG7 and varied from 0.43 ± 0.02 to 0.52 ± 0.02 and from 0.61 ± 0.02 to 0.65 ± 0.02 g/cm^3^ at D11 and YuMO, respectively. These values are different because of different sample holders and different powder compaction by tapping. We tended to measure and analyze the samples with the lowest density in order to avoid a contribution of multiple scattering. For the comparison of results, the powders were brought to the same density; the renormalization can lead to slight distortion.

**Figure 6:**
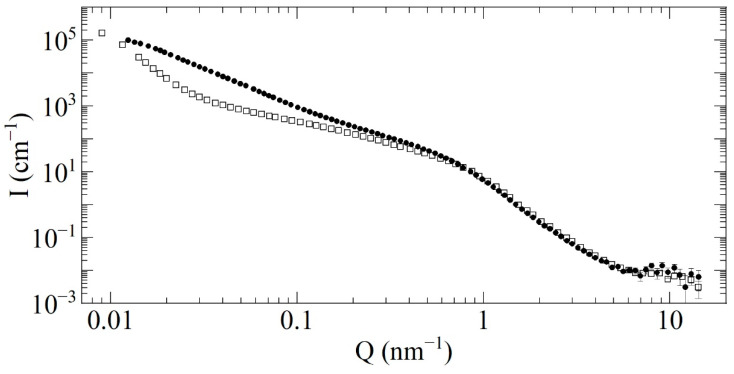
The intensity I (cm^−1^) of scattering versus the neutron-transferred momentum Q (cm^−1^) for the F-DNDs (solid circles) and DF-DNDs (open squares). Both curves are normalized to the equal sample density of 0.19 g/cm^3^.

The data from all instruments were merged to obtain a plot over the entire Q range, shown in Figure 6. The scattering curves for the samples of the two types largely differ only at small values of Q < 8·10^−1^ nm^−1^. Thus, the fraction of the agglomerates in DF-DNDs is much lower than it is in F-DNDs. The number of individual DNDs (corresponding to the large Q-values Q > 8·10^−1^ nm^−1^) increases by ~10% due to the destruction of agglomerates. Despite this slight difference in intensity, the shapes of two scattering curves are very similar. This observation is important for two reasons. First, it means that the size distribution of individual DNDs remains the same after the F-DNDs deagglomeration. Second, the incoherent scattering cross-section for both samples does not change, as evidenced by the equal intensity at Q > 7·10^0^ nm^−1^. Since for DNDs, this part of the cross-section is determined mainly by H, it implies that the fraction of H does not change after the F-DNDs deagglomeration. Both conclusions agree with our expectations based on the knowledge of the deagglomeration procedure.

## 4. Discussion

### 4.1. Size Distribution of Nanoparticles

The size distribution of DNDs were characterized with the optical methods, which do not contradict each other, but their results’ interpretation is somewhat ambiguous and is more of a complementary nature in relation to the neutron elastic scattering. The SANS data are more relevant for us; therefore, we used the model of discrete-sized diamond nanospheres to extract the size distribution of scatterers in the samples. The fitting procedure is stable against the initial parameter choice (populations, binning of radii, the radii range; it is wide enough). Here, we used 20 radii values per decade uniformly distributed on a logarithmic scale.

The radii range was selected based on the following arguments. The minimum radius is 0.6 nm; at this value, it is still energetically more advantageous for C atoms to form a diamond lattice [57]. The fraction of such particles was always equal to zero according to the fitting algorithm. The maximum radii are 200 nm for F-DNDs and 300 nm for DF-DNDs. These values describe the SANS data corresponding to the minimum transferred momentum Q_min_. They differ due to the different values of Q_min_ for the samples.

**Figure 7:**
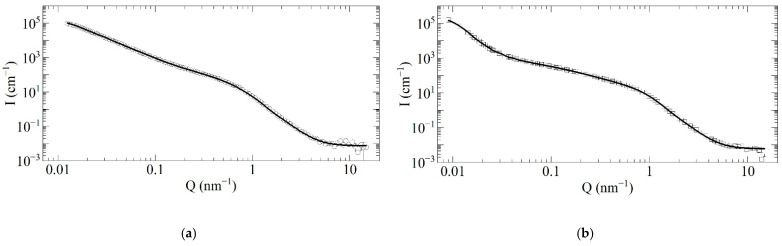
Comparison of measured (open circles and squares) and simulated (solid lines) scattering intensity I (cm^−1^) versus the neutron-transferred momentum Q (nm^−1^): (**a**) F-DND; (**b**) DF-DND samples.

Figure 7 shows measured and simulated SANS curves for both samples and demonstrates the validity of our model.

**Figure 8:**
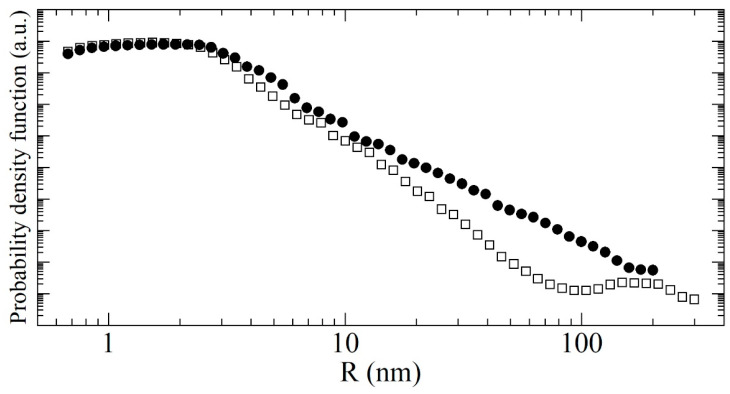
The probability density versus radius (in nm) calculated with the discrete-sized diamond nanospheres model for the F-DNDs (solid circles) and DF-DNDs (open squares). Points correspond to the results of calculation.

Figure 8 shows a log-log plot of the distribution of scatterer radii in both samples, extracted from the simulation curve of SANS data. One can see that the small radii dominate the distribution, and their amount does not significantly change with deagglomeration. More important for us is the decrease of the fraction of large clusters. It is small but dominates the neutron scattering in the low-Q region (shown in Figure 7).

**Figure 9:**
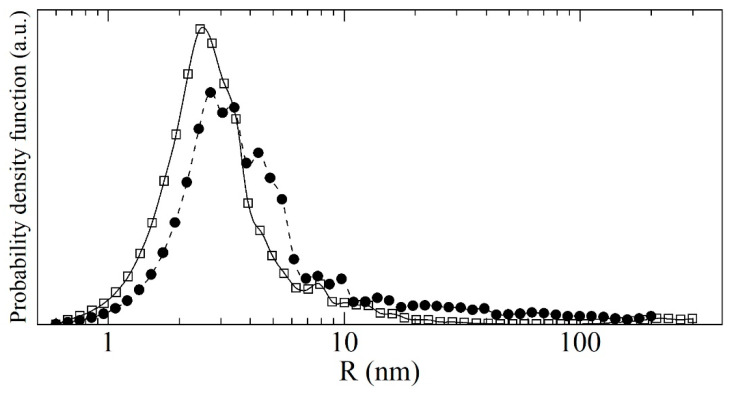
The log-linear volume distribution of scatterers evaluated using the discrete-sized diamond nanospheres model for the F-DNDs (solid circles) and DF-DNDs (open squares). Solid (DF-DNDs) and dashed (F-DNDs) lines interpolate the calculated results.

The radii distribution of DNDs from Figure 8 was transformed into the volumetric (mass) distribution of nanospheres (Figure 9), which can be directly compared with the results of non-neutron methods.

As one can see in Figure 9, the fraction of clusters with radii larger than 10 nm decreases in the case of DF-DNDs due to the deagglomeration. At the same time, the fraction of individual nanoparticles with a mean radius of 2.3 nm increases due to the destruction of agglomerates. In addition, we observe that the fraction of scatterers with radii 3–10 nm decreases. This can be due to the decrease of the fraction of individual nanoparticles sticking together: a neutron wave scattered coherently on such an object cannot distinguish it from a single particle of a larger size. On the other hand, such interpretation explains why the mean diameter of 4.7 nm for the individual nano-diamonds in F-DNDs decreases to the value of 4.0 nm after deagglomeration. However, the deagglomeration not only destroys the primary agglomerates but also causes formation of pores with sizes different from those present in F-DNDs. The neutron scattering on a pore structure is described by nanoparticles of a corresponding size that are also included in the resulting size distribution. It also might be a reason for the apparent decrease of the mean size of individual nanoparticles.

Extracted mean sizes of scatterers for different samples and radii ranges are as follows: in the radii range 0.6–10 nm, the mean size is 2.29 nm for F-DNDs and 1.97 nm for DF-DNDs; in the range 10–200 nm for F-DNDs and 10-300 nm for DF-DNDs, it is 16.2 nm and 13.4 nm, respectively. The most probable radius is 1.92 nm for F-DNDs and 1.53 nm for DF-DNDs.

Earlier, in [40], we introduced the effective mass of powder. Now, we supplement it by absolute calibrations of SANS data. Within this approach, it is defined as the ratio of the measured macroscopic cross-section of elastic coherent scattering to the analogous model cross-section. Non-diamond impurities decrease this factor, while some processes, like the inter-particle interference, might increase it. The evaluated effective masses are ~0.96 and ~0.86 for F-DNDs and DF-DNDs, respectively.

Another impact of the ongoing improvement of the procedure to extract the structural parameters is the difference between the current size distribution of F-DNDs and the previous one presented in [40]. Figure 8 shows the curves after the smoothing procedure we recently integrated into the fitting algorithm. These changes affect in no way the mean characteristics of the distributions and the description of SANS data.

### 4.2. Calculation of Albedo from a Flat Reflector

Using the SANS data presented in Section 3.5 and the model of interaction of neutrons with diamond nano-powders presented in Section 2.3, we simulated the albedo of neutrons from a semi-infinite and various finite-thickness media consisting of F-DNDs or DF-DNDs using the Monte Carlo method [58] and our original software [59]. For comparison, we present also results measured earlier for DNDs [40]. The density of F-DND and DF-DND powders and the composition of impurities absorbing neutrons are assumed to be the same. Incident neutrons are assumed to be isotropic.

**Figure 10:**
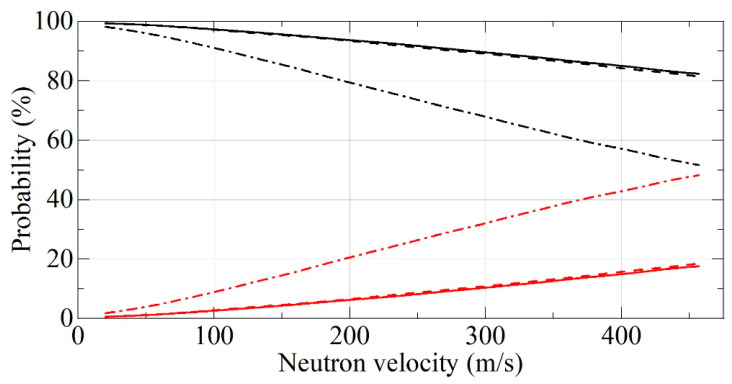
The probability of neutron reflection (black lines) and absorption (red lines) for F-DNDs (dashed lines) and DF-DNDs (solid lines) and DNDs (dash-dotted lines) versus neutron velocity. The incident neutrons are isotropic, the powder density is 0.19 g/cm^3^, and the powder thickness is infinite.

**Figure 11:**
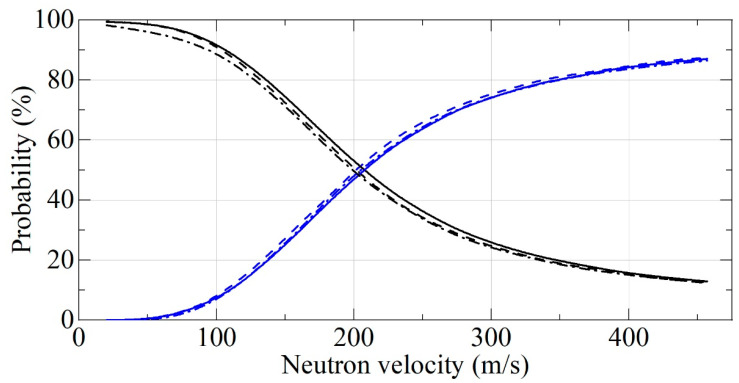
The probability of neutron reflection (black lines) and transmission (blue lines) for F-DNDs (dashed lines) and DF-DNDs (solid lines) and DNDs (dash-dotted lines) versus neutron velocity. The probability of neutron absorption is below 1% for F-DNDs and DF-DNDs at any velocity and 1–5% for DNDs. The incident neutrons are isotropic, the powder density is 0.19 g/cm^3^, and the powder layer thickness is 3 cm.

Figure 10 presents neutron albedo from the semi-infinite media, and Figure 11 shows an example of neutron albedo from a layer with a thickness of 3 cm. The statistical uncertainties of Monte Carlo simulations are smaller than the line thickness. We did not see a significant difference between F-DNDs and DF-DNDs albedo performance for the flat reflectors. As clear from Figure 10 for the semi-infinite media, both F-DNDs and DF-DNDs show a much better performance than that for DNDs in the total range of neutron velocities. On the other hand, Figure 11 shows similar performance for all materials for large neutron velocities; however, a much better performance of F-DNDs and DF-DNDs compared to that for DNDs for small neutron velocities. This is due to the fact that most of the neutrons with large velocities penetrate through the 3-cm-thick layer, and therefore, neutron losses are not really important in this case. On the other hand, neutrons with small velocities are reflected, and neutron losses during their long diffusion in powders is an important factor.

### 4.3. Calculation Storage Times in Closed Nano-Diamond Traps

While the performance of F-DNDs and DF-DNDs are similar for the flat reflector geometry, the situation changes dramatically when we consider a three-dimensional geometry of the reflector: an empty spherical cavity inside an infinite medium consisting of diamond nano-powder. To simulate neutron reflection in this geometry, we use the same SANS data and the same model of neutron interaction with powders.

**Figure 12:**
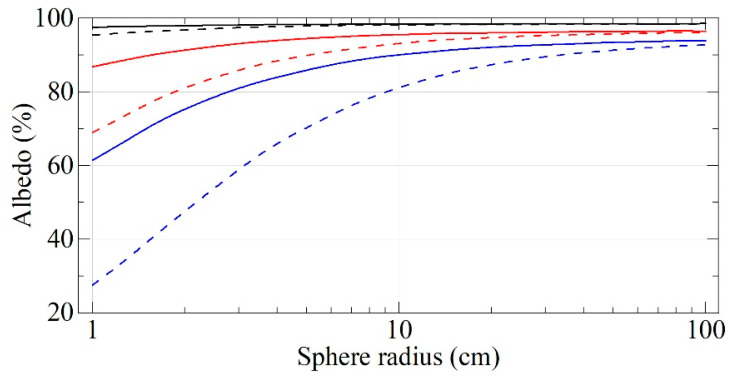
Neutron albedo for the velocities of 50 m/s (black lines), 100 m/s (red lines), and 150 m/s (blue lines) for F-DNDs (dashed lines) and DF-DNDs (solid lines) versus the cavity radius. The incident neutrons are isotropic, the powder thickness is infinite, and densities of F-DNDs and DF-DNDs are equal to 0.19 and 0.56 g/cm^3^, respectively.

**Figure 13:**
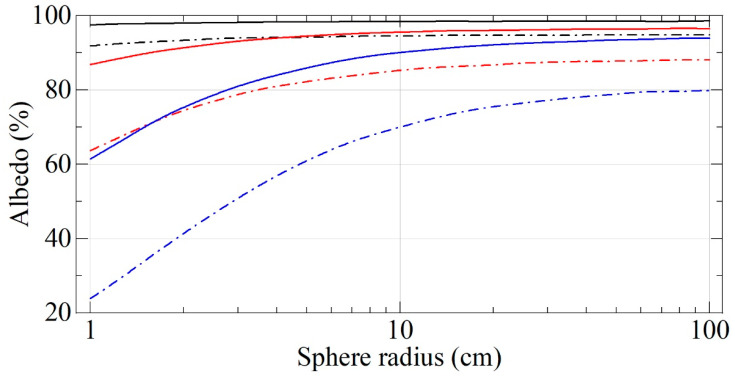
Neutron albedo for the velocities of 50 m/s (black lines), 100 m/s (red lines), and 150 m/s (blue lines) for DF-DNDs (solid lines) and DNDs (dash-dotted lines) versus the cavity radius. The incident neutrons are isotropic; the powder thickness is infinite. DF-DNDs density is equal to 0.56 g/cm^3^. The DNDs density is the same as it is in Figure 12 and equal to 0.19 g/cm^3^.

Figure 12 shows the albedo of neutrons from the cavity walls versus the neutron velocity and the cavity radius for both F-DND and DF-DND samples. For comparison, Figure 13 shows the albedo of neutrons from the cavity walls versus the neutron velocity and the cavity radius for DF-DND and DND samples. Note that a higher albedo value means a higher density of neutrons, which can be accumulated in the cavity.

DF-DNDs show much higher values than those for F-DNDs and DNDs for the entire range of neutron velocities and all cavity sizes. The gain over F-DNDs is primarily due to the higher density of the powder. Moreover, the greatest value of the gain factor is achieved in the case of approximate equality of the cavity radius and the depth of penetration of neutrons into the powder during reflection. The gain over DNDs is also due to the much smaller neutron losses due to the replacement of H with F.

## 5. Conclusions

We measured and simulated the effect of breaking agglomerates on the clustering of fluorinated diamond nanoparticles and on the efficiency of neutron reflectors, whose design is based on using the neutron albedo effect. We demonstrated that the reduction of clustering strongly decreases the scattering of neutrons with small momenta transfer and slightly increases the scattering of neutrons with large momenta transfer. The breaking of agglomerates is also evidenced by the results of DLS and SEM. The chemical composition, size distribution of individual nanoparticles, and IR spectra stay virtually unchanged. Our simulations show that the reduction of clustering increases neutron albedo and strongly increases neutron storage times in closed traps with DNDs walls, provided the neutron penetration depth is comparable to the trap size or the thickness of the trap wall. This effect is mainly due a significantly larger density of DF-DNDs than that of F-DNDs. These results are important for improving the efficiency of nano-diamond reflectors of slow neutrons.

## 6. Patents

The algorithm for extracting a model-independent size distribution of scatterers from small-angle scattering data has been developed for obtaining the results reported in this manuscript. It is protected by the author’s certificate of state registration of the software “Structural Nanopowders Analyzer Based on Small-Angle Scattering Data (SNASAS)” RU 2020662675 [60] issued by Federal Service for Intellectual Property.

## Data Availability

Neutron data were obtained in experiments at ILL, Grenoble, France: doi:10.5291/ILL-DATA.3-07-386, doi:10.5291/ILL-DATA.3-07-361.
